# Ischemic Ventricular Tachycardia Presenting as a Narrow Complex Tachycardia

**DOI:** 10.1016/s0972-6292(16)30777-x

**Published:** 2014-07-15

**Authors:** Stephen P Page, Troy Watts, Wee Tiong Yeo, Dhinoja Mehul

**Affiliations:** Department of Electrophysiology, St Bartholomew's Hospital, London, United Kingdom

**Keywords:** Ischemic Ventricular Tachycardia, Narrow Complex Tachycardia

## Abstract

This report describes a patient presenting with a narrow complex tachycardia in the context of prior myocardial infarction and impaired ventricular function. Electrophysiological studies confirmed ventricular tachycardia and activation and entrainment mapping demonstrated a critical isthmus within an area of scar involving the His-Purkinje system accounting for the narrow QRS morphology. This very rare case shares some similarities with upper septal ventricular tachycardia seen in patients with structurally normal hearts, but to our knowledge has not been seen previously in patients with ischemic heart disease.

## Introduction

Introduction Monomorphic ventricular tachycardia (VT) is commonly seen in patients with prior myocardial infarction and impaired left ventricular systolic function. In most cases of ischemic VT the QRS morphology during tachycardia is broad, as ventricular activation emerges from a critical isthmus of slow conduction and spreads away from the exit site passively without involvement of the specialized conduction tissue of the His-Purkinje system (HPS) [1]. In a minority of patients with ischemic VT however, HPS involvement can result in a relatively narrow QRS morphology - the majority of which have a right bundle branch block-like (RBBB) morphology and superior axis [2], similar to left posterior fascicular VT seen in patients with structurally normal hearts [3]. A re-entrant mechanism is usually identified and mapping of these tachycardias reveals a sharp Purkinje potential preceding the QRS complex at the successful ablation site [2]. We present a case of ischemic VT with a narrow QRS (implying HPS involvement) arising from an area of scar near the base of the left posterior septum with a mid-diastolic signal, concealed entrainment, and no Purkinje potential seen at the successful ablation site.

## Case Presentation

**Figure 1:**
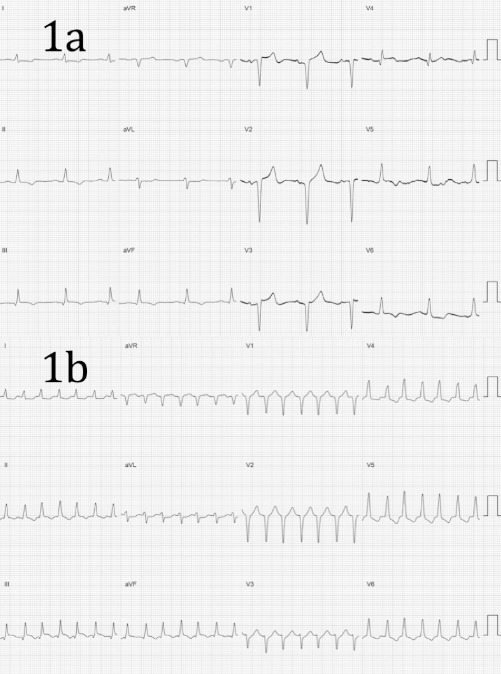
(a) 12-lead electrocardiogram in sinus rhythm. Paper speed 25mm/s. Gain 1mV = 10mm. (b) 12-lead electrocardiogram during tachycardia. Paper speed 25mm/s. Gain 1mV = 10mm.

A 62 year old man, with a history of remote inferior wall myocardial infarction, coronary artery bypass surgery and a dual chamber implantable cardioverter defibrillator (ICD) presented with multiple shocks related to recurrent ventricular tachycardia with a cycle length (CL) of 340 ms despite anti-arrhythmic drug therapy with sotalol. Coronary angiography revealed a chronic total occlusion of the right coronary artery, with moderate atheroma in the left coronary artery. An electrophysiology study was performed using the VelocityTM 3D mapping system (St Jude Medical, USA), a hexapolar catheter at the bundle of His, a decapolar catheter at the right ventricular apex and a roving irrigated-tip mapping catheter (NavistarTM Thermocool, Biosense-Webster Inc. CA, USA). A 12-lead electrocardiogram (ECG) in sinus rhythm showed Q waves in V1-3, a QRS duration of 102ms and T wave inversion in the inferolateral leads (Figure 1A).

**Figure 2:**
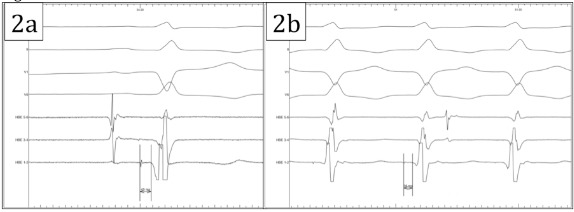
(a) His-Ventricular interval during sinus rhythm. Recordings from top to bottom are: surface ECG lead I, lead II, lead V1, lead V6, His 5-6, His 3-4 and His 1- 2. (b) His-Ventricular interval during tachycardia. Recordings from top to bottom are: surface ECG lead I, lead II, lead V1, lead V6, His 5-6, His 3-4 and His 1-2.

Baseline sinus intervals revealed an atrio-His (AH) and His-ventricular (HV) intervals of 132 and 45ms respectively (Figure 2A). Anterograde testing demonstrated smooth, decremental atrioventricular nodal conduction with no jump in the AH interval. Retrograde testing demonstrated decremental midline VA conduction. A narrow complex tachycardia (CL 340ms) was induced easily (and reproducibly) with programmed stimulation with a single extrastimulus from the right ventricle, with a QRS morphology which was very similar, but not identical, to the QRS morphology during sinus rhythm and a normal axis (Figure 1B). During tachycardia the small S wave in lead I was less prominent than during sinus rhythm and the small Q wave in V4 was not present. The intracardiac electrograms demonstrated ventriculo-atrial dissociation with a His signal preceding every ventricular electrogram with a HV interval of 36ms (shorter than during sinus rhythm (Figure 2B)).

His-synchronous ventricular premature beats did not advance the subsequent His signal, it was not possible to entrain the tachycardia from the atria, and the tachycardia cycle length did not increase with the development of catheter induced right bundle branch block. Activation mapping localized the earliest ventricular signal to the base of the left ventricle approximately 2 cm below the His catheter within the border zone of a large area of scar on the inferior left ventricular wall (Figure 3a). A mid-diastolic signal was identified in this area and pacing from this site was associated with concealed entrainment (Figure 3b).

Radiofrequency energy was delivered at this site with termination of the tachycardia after 16 seconds. No Purkinje potential was recorded at this site either during tachycardia or during sinus rhythm. The tachycardia was non-inducible with programmed stimulation subsequently. The patient made an uneventful recovery and remains well 6 months later with no recurrence of ventricular tachycardia.

## Discussion

**Figure 4:**
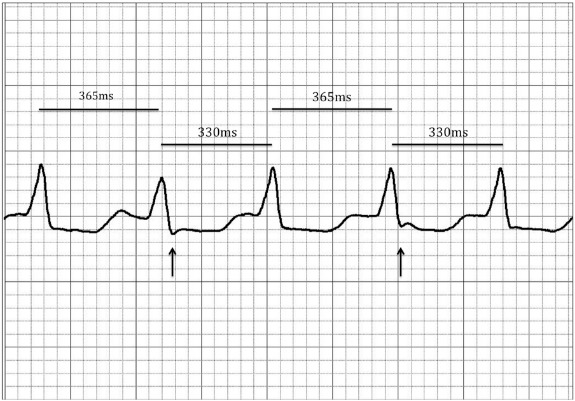
Occasional alternans in the tachycardia cycle length was observed and associated with beat to beat variation in the QRS morphology suggesting degrees of fusion of myocardial activation.

We have described a patient with ischemic heart disease presenting with VT with a narrow QRS morphology in whom the successful ablation target at the posterior basal septum revealed a low amplitude, mid-diastolic signal and no Purkinje potential. The QRS morphology during tachycardia was almost identical to that seen during sinus rhythm except for a subtle change in the polarity of limb lead I and V4. This suggests that the majority of ventricular activation occurred via the His-Purkinje system with a small degree of fusion accounting for the subtle differences between tachycardia and sinus rhythm. In fact subtle alternans in the cycle length was noted occasionally during tachycardia and was associated with beat to beat variation in the presence of the small S wave in limb lead I (Figure 4).

Ventricular tachycardia with a relatively narrow QRS morphology is well recognized in patients with idiopathic fascicular VT of which there are three main types: (1) left posterior fascicular VT with RBBB and superior axis, (2) left anterior fascicular VT with RBBB and inferior axis and (3) the very rare upper septal VT with a normal QRS morphology and normal axis [3]. The VT observed in our patient has several similarities with upper septal fascicular VT [3-5]. Firstly, the QRS morphology during tachycardia was narrow and very similar to that seen during sinus rhythm. Secondly, the successful ablation site was localized to the basal septum of the left ventricle, and thirdly the HV interval during tachycardia was shorter than during sinus rhythm. Unlike previous reports however, the successful ablation site did not reveal a pre-systolic Purkinje potential, but a fractionated, low amplitude, mid-diastolic potential which could be entrained with concealed fusion.

In patients with structural heart disease involvement of the His-Purkinje system during ventricular tachycardia is uncommon, but well recognized [2]. Lopera et al described 20 patients with structural heart disease and His-Purkinje system (HPS) involvement [6]. Of these 16 had typical bundle branch re-entry (BBR), 2 had BBR and inducible interfascicular VT and 2 had a focal automatic tachycardia in the distal HPS. In the two patients with a focal automatic tachycardia the QRS morphology during tachycardia was similar to that during sinus rhythm (as in our patient). Bogun et al described 9 patients with ischemic VT, a relatively narrow QRS (≤145ms) and HPS involvement but excluded BBR, interfascicular re-entry and fascicular VT as a mechanism [2]. Of the 9 patients, 7 had a RBBB and left axis morphology, one a RBBB and right axis morphology and in one a LBBB morphology (each different from the QRS morphology in sinus rhythm). In each patient they demonstrated a re-entrant mechanism and identified a Purkinje potential at each successful ablation site.

Hayashi et al. described 4 patients with prior myocardial infarction in whom VT involving the His-Purkinje system was observed [7]. In all cases the QRS morphology during tachycardia was different from sinus rhythm and the successful ablation site in the mid-ventricular posterior septum was associated with a Purkinje potential in tachycardia and sinus rhythm.

Sakamoto et al. described a patient with previous inferior myocardial infarction in whom a relatively narrow complex tachycardia (QRSd 120ms) was induced which was similar but not identical to the QRS morphology in sinus rhythm [8]. In this patient the successful ablation site was localized to the posteroseptal mitral annulus. The authors believed that the HPS was not involved on the basis that no His signal was recorded during tachycardia although it is possible that the His signal was buried within the ventricular electrogram.

The clinical significance of this case is that VT with HPS involvement (and a tachycardia QRS morphology almost identical to sinus rhythm) should occasionally be considered in the differential diagnosis of a narrow complex tachycardia. In our patient VA dissociation was observed facilitating the recognition of VT, although 1:1 VA association would certainly be possible if retrograde VA conduction were sufficiently rapid. The differential diagnosis of a narrow complex tachycardia with VA dissociation includes junctional tachycardia with retrograde block, AV nodal reentry tachycardia (AVNRT) with upper common pathway block, orthodromic AV reentry tachycardia (AVRT) associated with a nodofascicular accessory pathway as the retrograde limb of the circuit and upper septal fascicular VT. Differential pacing during tachycardia and the location of the successful ablation site excluded all of the above possibilities however, leaving VT with HPS involvement as the best explanation for our findings.

**Figure 5:**
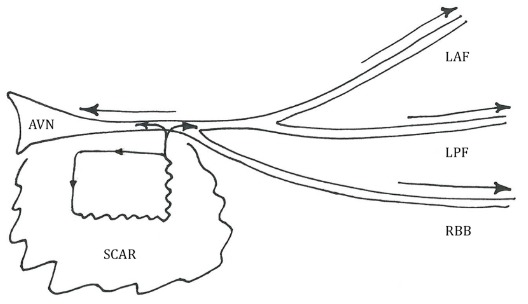
AVN - atrioventricular node, LAF - left anterior fascicle, LPF - left posterior fascicle, RBB - right bundle branch. The proposed mechanism involves microreentry within an area of scar inferior to the His bundle. Activation of the proximal His-Purkinje system is shown by the arrow heads.

We cannot be certain of the precise mechanism of this tachycardia, however reentry is likely given that the tachycardia was easily and reproducibly initiated with programmed stimulation from the ventricle, the tachycardia could be reproducibly entrained, and there was a mid-diastolic signal at the successful ablation site suggesting a critical isthmus involving, or close to, the HPS in the region of the posterior basal LV septum. Activation mapping identified a localized focal area of earliest activation suggesting that micro-reentry was the likely mechanism. We assume that the narrow QRS morphology and nearly identical morphology to sinus rhythm is due to the involvement of the proximal HPS with balanced anterograde activation of all the main branches of the HPS. The proposed mechanism of this tachycardia is shown in Figure 5. The subtle change in QRS morphology on a beat to beat basis suggest subtle degrees of fusion of myocardial activation. Whether the HPS in the posterior basal septum is critical to the circuit or is within a protected area of scar and activated passively is impossible to determine.

In summary we have described a patient with scar-related ischemic VT with a narrow QRS morphology with a mechanism different to that normally seen in this context. The presence of a mid-diastolic potential and the absence of a pre-systolic Purkinje potential is unusual and the nearly identical QRS morphology to sinus rhythm is very unusual for VT and might lead to an erroneous diagnosis of supraventricular tachycardia unless alert to the possibility of this rare form of scar-related VT.

## Figures and Tables

**Figure 3a:**
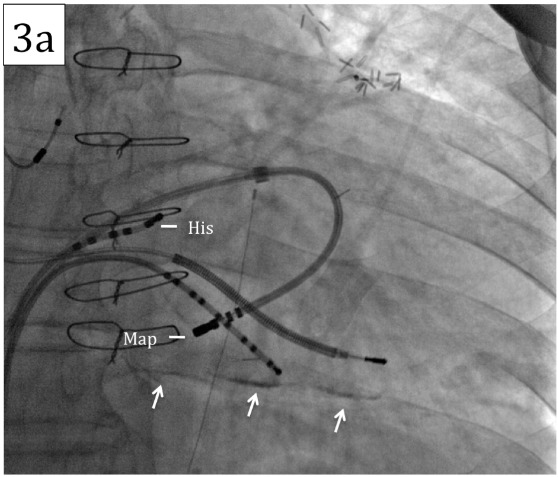
Fluoroscopy of catheter position at successful ablation site. Note thepresence of calcium within the scarred inferior wall (arrows). The position of the His catheter and mapping catheter are marked.

**Figure 3b:**
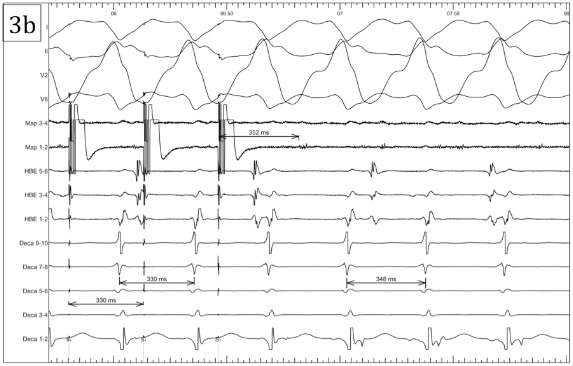
Entrainment mapping at the successful ablation site leading to termination of the tachycardia. The tachycardia was entrained with concealed fusion pacing from the distal poles of the mapping catheter at 330ms. The post-pacing interval (352ms) minus the return cycle length (348ms) was 4ms. Of note, the His signal was not apparent in this recording - attributed to micro-displacement of the His catheter and the timing of the ventricular electrograms recorded by the His catheter in relation to QRS onset are delayed in Fig 3b compared to Fig 2b reflecting the development of catheter induced right bundle branch block.

**Figure 3c:**
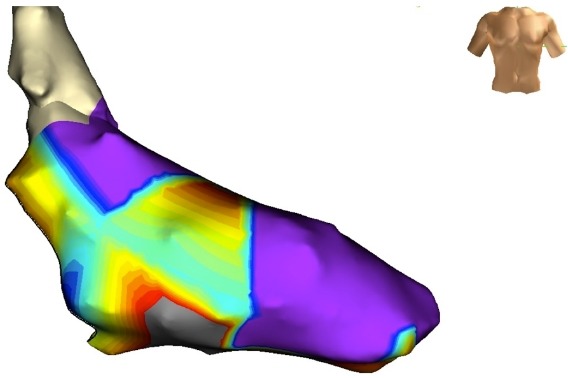
3D voltage map of the left ventricle. Bipolar voltage < 0.5mV was designated as scar (grey) and normal voltage as > 1.5mV (purple) with areas of intermediate voltage (yellow/green/blue).
